# Supplementary material for: Vitamin A Plasma Levels in COVID-19 Patients: A Prospective Multicenter Study and Hypothesis
Source: Nutrients. 2021 Jun 24;13(7):2173. doi: 10.3390/nu13072173 (PMC8308355; doi:10.3390/nu13072173)
Supplement: Supplementary file 1 [file nutrients-13-02173-s001.zip › nutrients-1257621-supplementary.pdf]

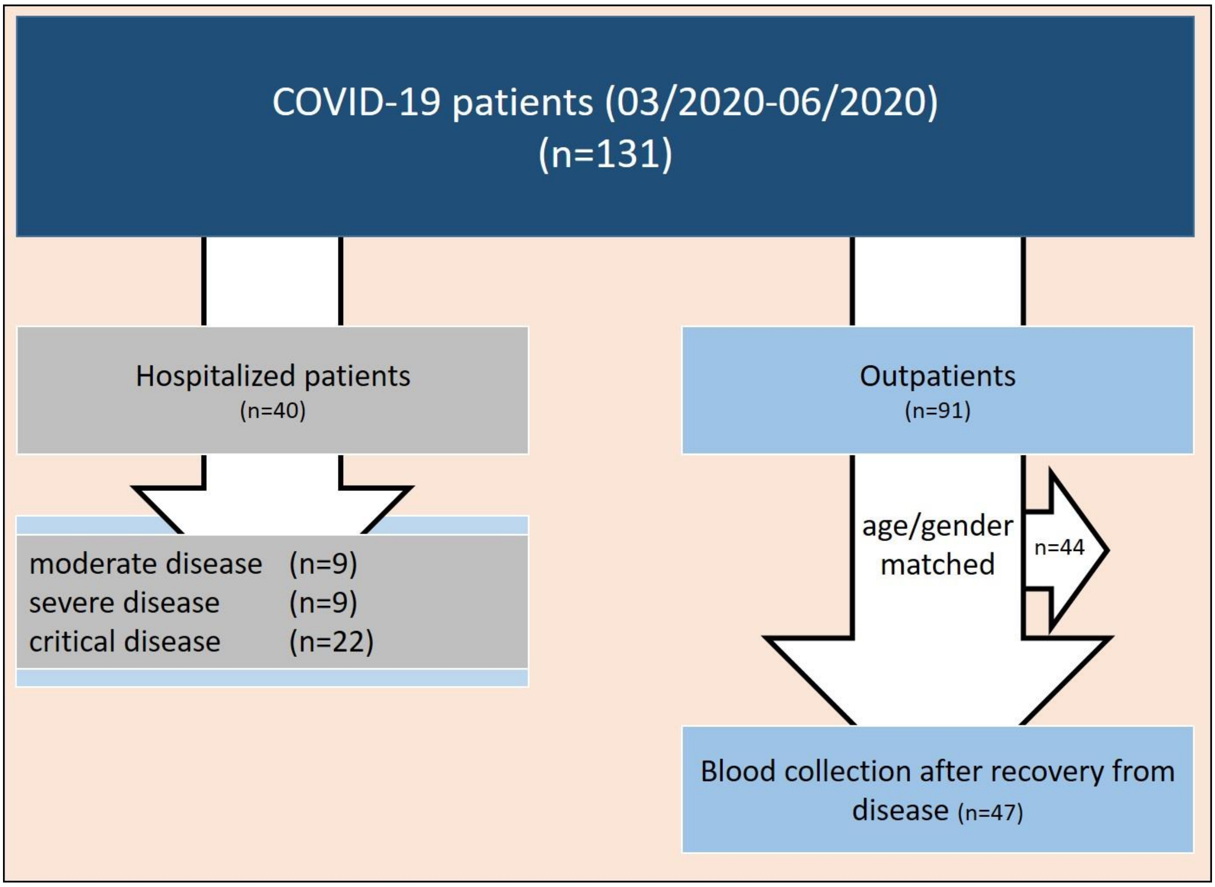

Supplementary Figure S1. Study flowchart Vitamin A plasma levels in COVID-19 patients: A prospective multicenter study and hypothesis. COVID-19, coronavirus disease 2019.

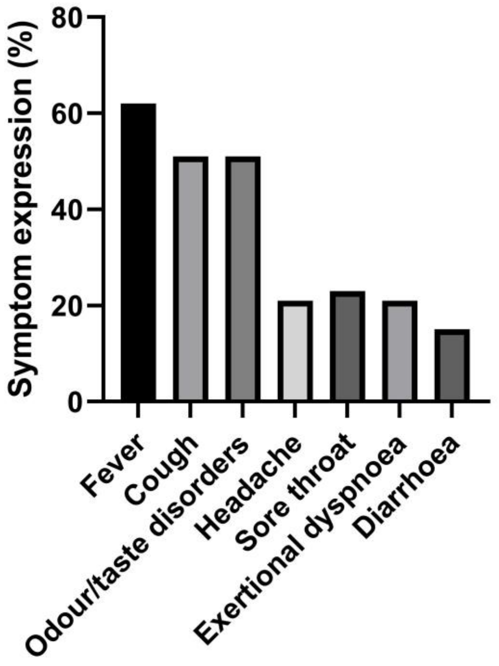

Supplementary Figure S2. Representation of the percentage symptom expression of the convalescent COVID-19 patient cohort during acute phase of disease. At the time of blood sampling after recovery from disease, the patients were all free of symptoms.

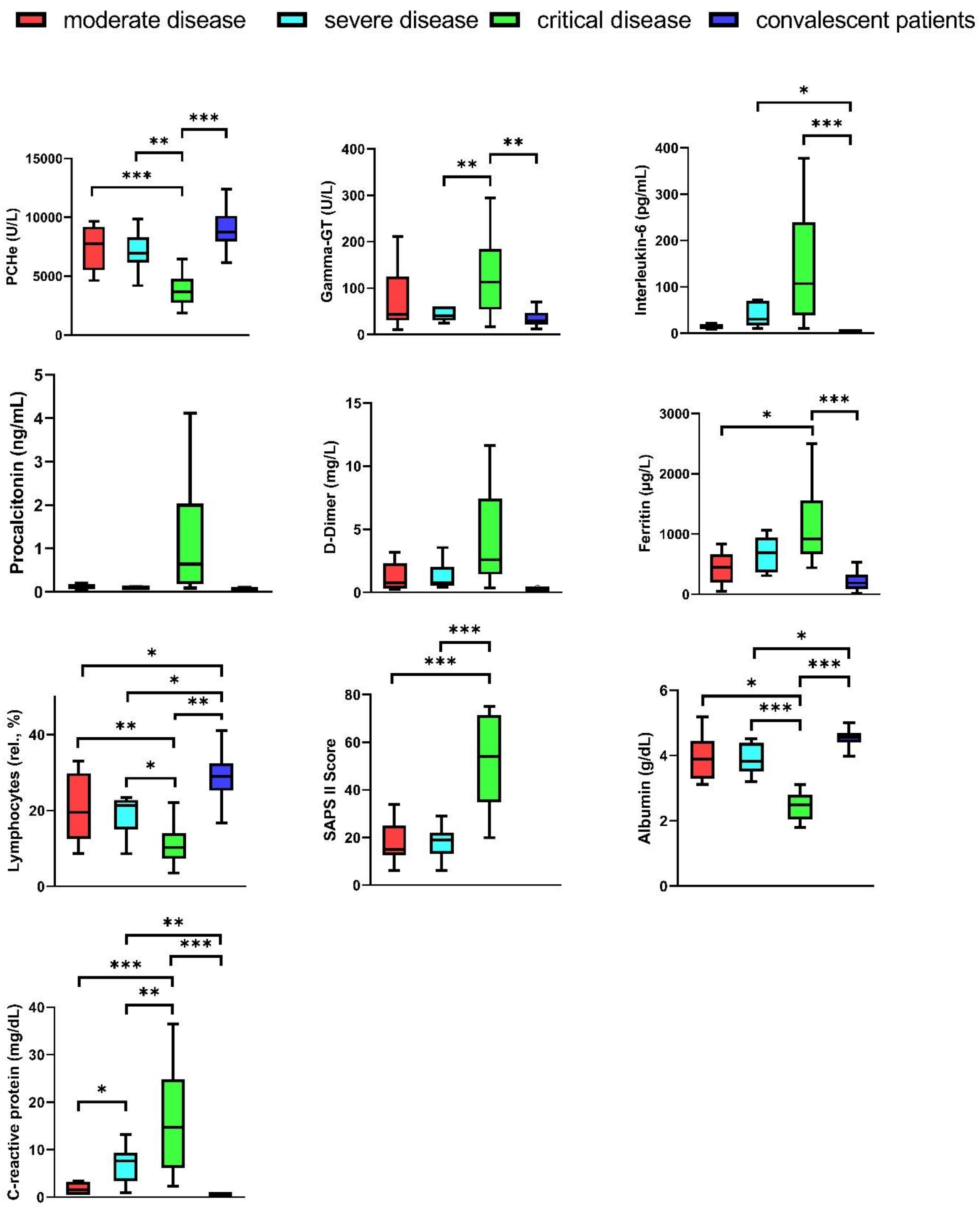

Supplementary Figure S3. Presentation of important laboratory parameters. Classification into moderate, severe and ciritical disease and convalescent patients (*$p < 0.05$, **$p < 0.01$, ***$p < 0.001$).
